# Features of Lipid Metabolism in Humanized ApoE Knockin Rat Models

**DOI:** 10.3390/ijms22158262

**Published:** 2021-07-31

**Authors:** Yang Wu, Gem Johnson, Fujie Zhao, Yin Wu, Guojun Zhao, Andrew Brown, Shaojin You, Ming-Hui Zou, Ping Song

**Affiliations:** 1 Center for Molecular and Translational Medicine, Georgia State University, Atlanta, GA 30303, USA; ywu13@gsu.edu (Y.W.); gjohnson90@student.gsu.edu (G.J.); fzhao@gsu.edu (F.Z.); wuyin0923@126.com (Y.W.); syou2@gsu.edu (S.Y.); mzou@gsu.edu (M.-H.Z.); 2 Envigo RMS, Inc., St. Louis, MO 63146, USA; guojun.zhao@envigo.com (G.Z.); andrew.brown@envigo.com (A.B.)

**Keywords:** ApoE isoform, rat model, lipid metabolism, dyslipidemia, Paigen diet, vascular remodeling

## Abstract

Apolipoprotein E (ApoE), an essential plasma apolipoprotein, has three isoforms (E2, E3, and E4) in humans. E2 is associated with type III hyperlipoproteinemia. E4 is the major susceptibility gene to Alzheimer’s disease (AD) and coronary heart disease (CHD). We investigated lipid metabolism and atherosclerotic lesions of novel humanized ApoE knockin (hApoE KI) rats in comparison to wide-type (WT) and ApoE knockout (ApoE KO) rats. The hApoE2 rats showed the lowest bodyweight and white fat mass. hApoE2 rats developed higher serum total cholesterol (TC), total triglyceride (TG), and low- and very low density lipoprotein (LDL-C&VLDL-C). ApoE KO rats also exhibited elevated TC and LDL-C&VLDL-C. Only mild atherosclerotic lesions were detected in hApoE2 and ApoE KO aortic roots. Half of the hApoE2 rats developed hepatic nodular cirrhosis. A short period of the Paigen diet (PD) treatment led to the premature death of the hApoE2 and ApoE KO rats. Severe vascular wall thickening of the coronary and pulmonary arteries was observed in 4-month PD-treated hApoE4 rats. In conclusion, hApoE2 rats develop spontaneous hyperlipidemia and might be suitable for studies of lipid metabolism-related diseases. With the PD challenge, hApoE4 KI rats could be a novel model for the analysis of vascular remodeling.

## 1. Introduction

Apolipoprotein E (ApoE) is one of the most important plasma apolipoproteins involved in the metabolism of fats. It is synthesized and secreted from a variety of tissues, including the liver, brain, adipose tissue, adrenal gland, muscle tissue, arterial wall, and monocytes/macrophages, but is derived primarily from the liver [1,2]. ApoE is present as part of triglyceride-rich lipoproteins (very low density lipoproteins (VLDL-C), intermediate-density lipoproteins (IDL), and chylomicron remnants) and high-density lipoproteins (HDL-C) and interacts significantly with a number of members of the low-density lipoprotein receptor (LDLR) family, such as the LDL receptor-related protein 1 (LRP1), the VLDL receptor, and the ApoE2 receptor (LPR8) [3,4]. ApoE interacts with these receptors and other proteins promoting the endocytic clearance of blood lipoproteins. Besides lipid transportation, this protein is also involved in non-lipid-related functions, including glucose homeostasis, immunoregulation, oxidation, and cell proliferation and migration [5,6]. Human ApoE has three isoforms (E2, E3, and E4). ApoE3 is the most common one, with a population allelic frequency of 78% [7], and is considered to be the normal form. The ApoE2 isoform, with the lowest allelic frequency (7%), differs from ApoE3 by the single amino acid substitution Arg158Cys which disrupts the nearby LDLR recognition site, exhibiting very low binding affinity to LDLR and impaired clearance of triglyceride-rich lipoprotein remnant particles [8]. ApoE2 is associated with the genetic disorder type III hyperlipoproteinemia (higher cholesterol and triglyceride levels) [9]. ApoE4 has an allelic frequency of 15% and differs from ApoE3 by the substitution Cys112Arg. This isoform has an intact binding affinity to LDLR but enhanced lipid-binding ability to large triglyceride-rich VLDL-C particles [8,10]. This may lead to downregulation of LDLR and impaired lipolytic processing in the circulation. ApoE4 is the major susceptibility gene related to the onset of Alzheimer’s disease [11,12] and also associates with dyslipidemia [13,14] as well as coronary heart disease (CHD) [15,16].

The generation of mice carrying a mutant ApoE gene or different ApoE isoforms, such as ApoE knockout (ApoE KO) and humanized ApoE knockin (hApoE KI) mice [17,18,19,20,21], provides powerful tools for studies of lipid metabolism, Alzheimer’s disease, and cardiovascular diseases (CVDs), including atherosclerosis and CHD. Although mouse models are widely used, they are not able to fully recapture all of the human physiology or resemble all human diseases, especially in neuroscience and behavioral studies [22]. Additionally, the small size of mice limits the surgical procedures and accuracy in measurement of the experimental lesions as well as drug administration [23]. Rat models are better than mice in these aspects while keeping the advantages of ease of maintenance, a short breeding time, and economic savings. Furthermore, it is also necessary to validate the translational value of mouse studies to human diseases in other species. ApoE-deficient rat models generated by research labs and Envigo RMS (HsdSage:SD-*ApoE^em1Sage^* rat, Saint Louis, MO, USA) have been used in studies of atherogenic phenotypes and Alzheimer’s disease [24,25,26,27]. hApoE KI rat models with a replacement of the entire endogenous rat ApoE with the human ApoE2, E3, or E4 alleles have become available recently at Envigo RMS. Only one study has been performed on characterizing the brain structure alternations and behavior changes in hApoE4 rats [28]. The lipid metabolism features and vascular phenotypes of these hApoE KI rats remain unknown. Here, we investigated the phenotypes of these hApoE KI rats in comparison to WT and ApoE KO rats, as well as the lipid metabolic changes and vascular remodeling of them after a Paigen diet (PD) challenge.

## 2. Results

### 2.1. Expression of Rat ApoE and Human ApoE in WT, hApoE KI, and ApoE KO Rats

**Figure 1:**
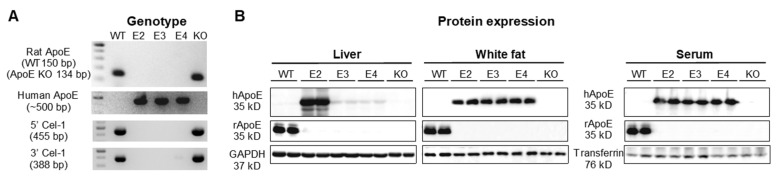
ApoE expressions in transgenic hApoE KI rats. (**A**) Genotyping was performed to detect the genetic constitution of different rats. The ApoE knockout (KO) rat was generated by a 16 bp deletion at 1434–1449 of the ApoE gene. (**B**) Protein expressions of rat or human ApoE in the liver, subcutaneous white fat, and serum were detected by Western blot with specific antibodies. WT, wild type; E2, hApoE2; E3, hApoE3; E4, hApoE4.

We confirmed the ApoE genotype and expression by PCR and Western blot analysis (Figure 1). Genomic DNA of the rats was isolated from rat tail or ear tissue, and genotyping with specific primers was performed (Figure 1A). ApoE protein expression in the liver, subcutaneous white fat, and serum was detected by anti-rat and anti-human ApoE antibodies (size~35 kDa) (Figure 1B). hApoE KI rats exclusively expressed human ApoE proteins, while WT rats exclusively expressed the rat ApoE protein. Fat tissue and serum collected from different hApoE KI rats showed similar expression levels of the human ApoE protein. However, hApoE2 livers expressed a much higher level of human ApoE in comparison to hApoE3 and hApoE4 livers.

### 2.2. Bodyweight, Food Consumption, and Fat Mass

**Figure 2:**
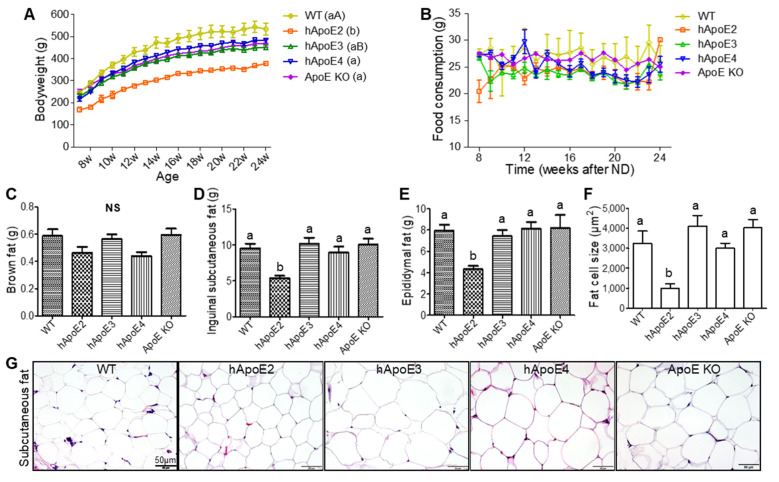
Bodyweight, food consumption, and fat mass of WT, hApoE KI, and ApoE KO rats. (**A**). Bodyweights of different rats fed with ND were monitored every week from age 7 to 24 weeks. (**B**) Food intakes per day of the same rats were recorded every week as well. Tissue weights of brown adipose tissue (**C**), inguinal subcutaneous fat (**D**), and epididymal fat (**E**) were measured after euthanizing animals at age 6 months. (**F**) Size of inguinal subcutaneous adipocytes of WT, hApoE, and ApoE KO rats was quantitatively analyzed. (**G**) Representative photomicrographs of inguinal adipocytes with HE stain were compared with 4 to 5 µm paraffin sectioning. (Data represent mean values ± SEM. WT n = 9; hApoE2 n = 10; hApoE3 n = 11; hApoE4 n = 11; ApoE KO n = 6. One-way ANOVA followed by a Tukey post hoc test. a vs. b, A vs. B, *p* < 0.05, NS, no significant difference).

We firstly investigated the rats’ bodyweight starting from age 7 weeks and found that all the hApoE KI and ApoE KO rats had a significantly lower bodyweight in comparison to WT rats at the corresponding age (Figure 2A). Among the transgenic rats, hApoE2 rats showed the lowest bodyweight. When the animals got older, such differences became bigger (Figure 2A). Food consumption showed no significant difference among these rats (Figure 2B). We checked the fat mass and found that the mass of the inguinal subcutaneous fat (Figure 2D) and epididymal fat (Figure 2E), but not the back brown adipose tissue (Figure 2C), was lower in hApoE2 rats. Meanwhile, the fat masses of ApoE KO rats showed no difference from those of WT, hApoE3, and E4 rats (Table 1). The adipocyte size of the hApoE2 subcutaneous fat was also smaller compared to that of other rats (Figure 2F,G). These data suggest reduced lipid storage in the adipose tissue and the smaller adipocytes of the hApoE2 rats.

### 2.3. Serum Lipid and Glucose Metabolism Profiles

**Figure 3:**
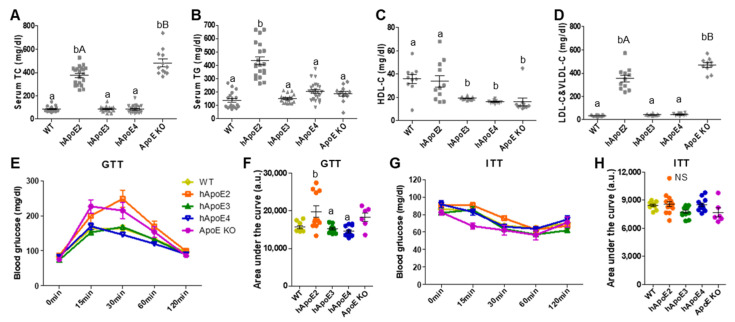
Metabolism sketch of WT, hApoE KI, and ApoE KO rats. (**A**–**D**) The levels of TC, TG, HDL-C, and LDL-C&VLDL-C in serum of each rat strain collected at age 8 weeks were detected after overnight fasting (~18 h) (TC and TG: WT n = 18, hApoE2 n = 20, hApoE3 n = 20, hApoE4 n = 23, and ApoE KO n = 12; HDL-C, and LDL-C&VLDL-C: WT n = 10, hApoE2 n = 11, hApoE3 n = 12, hApoE4 n = 10, and ApoE KO n = 10). (**E**,**F**) GTT with i.p. injection of 2 g glucose per kg of bodyweight was performed on rats at age 12 to 14 weeks after overnight fasting (WT n = 9, hApoE2 n = 11, hApoE3 n = 11, hApoE4 n = 12, and ApoE KO n = 6). (**G**,**H**) ITT with i.p. injection of 0.75 U insulin per kg of bodyweight was performed on rats at the same age after 6-h fasting. (One-way ANOVA followed by a Tukey post hoc test. a vs. b, A vs. B, *p* < 0.05, NS, no significant difference).

As shown in Figure 3A–D, hApoE2 rats developed spontaneous hyperlipidemia. At age 8 weeks, the TC, TG, and atherogenic lipoprotein LDL-C&VLDL-C levels of hApoE2 rats were all significantly higher compared to those of WT, hApoE3, and hApoE4. ApoE KO rats exhibited higher levels of TC and LDL-C&VLDL-C than hApoE2 rats (Figure 3A,D), but not higher TG levels (Figure 3B). The hApoE3 and hApoE4 rats had lower anti-atherogenic lipoprotein HDL-C (Figure 3C). Such lipid levels maintained a similar trend at age 6 months (Figure 7A–D, ND groups).

Lipid metabolism and glucose metabolism are associated with each other in many ways. We found that fasting glucose levels were not influenced by ApoE isoforms or ApoE deficiency (Figure 3E, 0 min pots). GTT suggested a slightly impaired glucose tolerance of hApoE2 rats at age 12 to 14 weeks in comparison to hApoE3 and hApoE4 rats (Figure 3E,F). When the animals got older (at age 6 months), no significant difference was detected among WT and hApoE KI rats (Figure 7E,F). No significant difference in the insulin response was observed among WT, hApoE KI, and ApoE KO rats at age 3 months or 6 months when analyzed with ITT (Figure 3G,H and Figure 7G,H).

### 2.4. Ectopic Lipid Deposition In Vital Organs and Hepatic Nodular Cirrhosis in hApoE2 Rats

**Figure 4:**
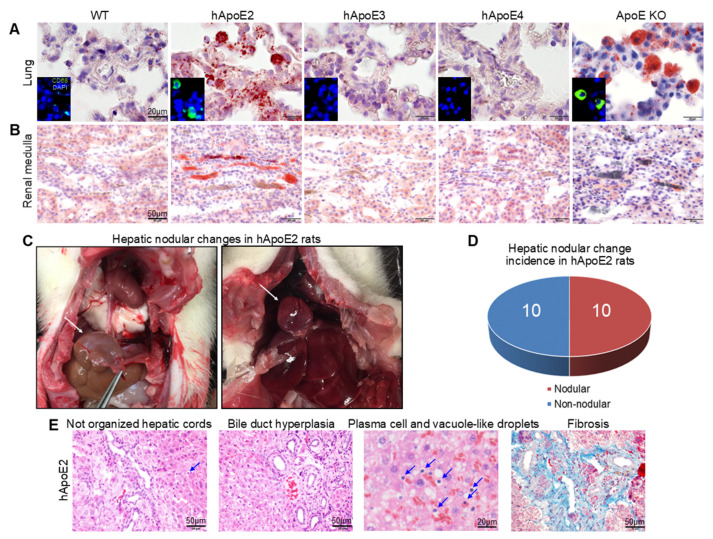
Increased lipid deposition and hepatic nodular cirrhosis in hApoE2 rats. (**A**) Oil Red O staining was used to assess the neutral fat deposit in rat lungs with 8 µm frozen sectioning. Increased Oil Red O-stained cells were detected in the lung tissue of hApoE2 and ApoE KO rats. Anti-CD68 antibody stained these lipid-laden cells. (**B**) Fat deposit in renal medulla was stained by Oil Red O staining. Red blood cells differentiate vessels from tubules in the renal medulla. (**C**) The hepatic nodular tissue of hApoE2 rats was located on the top of the liver and clinging to the diaphragm. Some of the hepatic nodular tissue even bumped into the chest. White arrow indicates the hepatic nodular tissue. (**D**) Incidence of hepatic nodular change in hApoE2 rats is indicated. (**E**) The histological abnormalities in hepatic nodular tissue of hApoE2 showed changes in hepatic nodular cirrhosis, including hepatic cords without the classical radial structure, bile duct hyperplasia, increased plasma cells (blue arrow), vacuole-like droplets (lipid droplets after staining processing), and hepatic fibrosis. The hepatic structure in areas other than nodular change is presented in Figure S1E. Masson staining was used to assess fibrosis, and HE staining was used for the rest of the slides.

Both hApoE2 and ApoE KO rats exhibited hyperlipidemia when fed with the normal diet. High blood lipids may lead to ectopic lipid deposition in cells and organs. Oil Red O was used to demonstrate the presence of fat or lipids in formalin-prefixed tissues. Increased lipid accumulation was observed in the lungs of hApoE2 and ApoE KO rats by Oil Red O staining (Figure 4A). Specific immunofluorescence staining with the anti-CD68 antibody suggested the lipid-laden cells in hApoE2 lungs were macrophages. Ectopic lipid deposition was detected in the renal medulla of hApoE2 rats, possibly in the forms of fatty casts in renal collecting ducts and lipid droplets accumulated in the renal vessels (Figure 4B). Meanwhile, the heart, liver, and renal cortex of hApoE2 rats did not have ectopic lipid deposition (Figure S1A–C). It seems that lipids deposit macrophages of hApoE2 and ApoE KO lungs easily.

A high blood lipid level is an important risk factor for the development of liver diseases such as non-alcoholic fatty liver disease, non-alcoholic steatohepatitis, and even cirrhosis [29,30]. Therefore, we investigated the liver structure of all hApoE KI and ApoE KO rats in comparison to WT rats. The age-matched liver mass did not show a significant difference (data not shown). We found a nodular change in the liver of hApoE2 rats (white arrow indicated in Figure 4C and Figure S1D). All these nodular hepatic tissues in hApoE2 livers were singular and closely attached to the diaphragm. Some big ones even protruded into the chest cavity (Figure 4C right pattern). The incidence of such liver change is 50% (10 out of 20 hApoE2 rats) (Figure 4D). Liver tissue outside of the hApoE2 nodule showed a relatively normal histological morphology (Figure S1E), while all nodular hepatic tissues of hApoE2 exhibited loss of the normal lobule hepatic structure, bile duct hyperplasia, increased plasma cells, mild steatosis, and increased fibrosis (Figure 4E). These changes in the hApoE2 rat livers suggest hepatic nodular cirrhosis.

### 2.5. Mild Atherosclerotic Lesion in hApoE2 Rats

**Figure 5:**
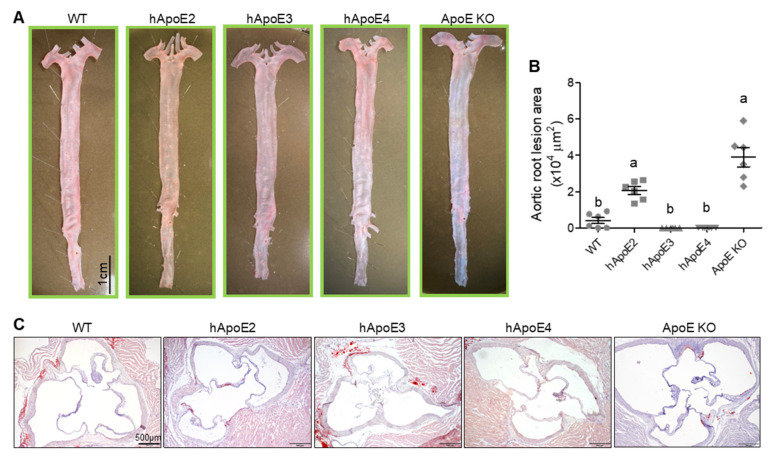
Mild atherosclerotic plaque formation in hApoE2 and ApoE KO rats. (**A**) Representative photographs of Oil Red O-stained en face aortas from rats aged 6 months are shown. (**B**) Plaque lesion areas in cross-sectional aortic roots of different rats were quantified at age 6 months. (n = 6 per group, One-way ANOVA followed by a Tukey post hoc test. a vs. b, significant difference *p* < 0.05). (**C**) Representative photomicrographs of Oil Red O-stained cross-sectional aortic roots are shown.

Rats and mice appeared to be highly resistant to atherosclerosis due to the low levels of pro-atherogenic plasma lipoproteins LDL-C and VLDL-C and high levels of anti-atherosclerotic HDL-C [31,32,33,34]. The mutation or deficiency of the rat ApoE protein could accelerate the formation of atherosclerotic plaques. Therefore, we investigated atherogenesis in these hApoE KI rats. Surprisingly, although hApoE2 and ApoE KO rats developed spontaneous high blood lipid levels, no obvious aortic plaques were identified in their en face aortas at age 6 months (Figure 5A). Only mild atherosclerotic lesions were detected in the aortic roots of hApoE2 and ApoE KO rats (Figure 5B,C). The lesion area of ApoE KO rats was larger than that of hApoE2 rats.

### 2.6. Premature Death of hApoE2 and ApoE KO Rats after a Short Period of PD

**Figure 6:**
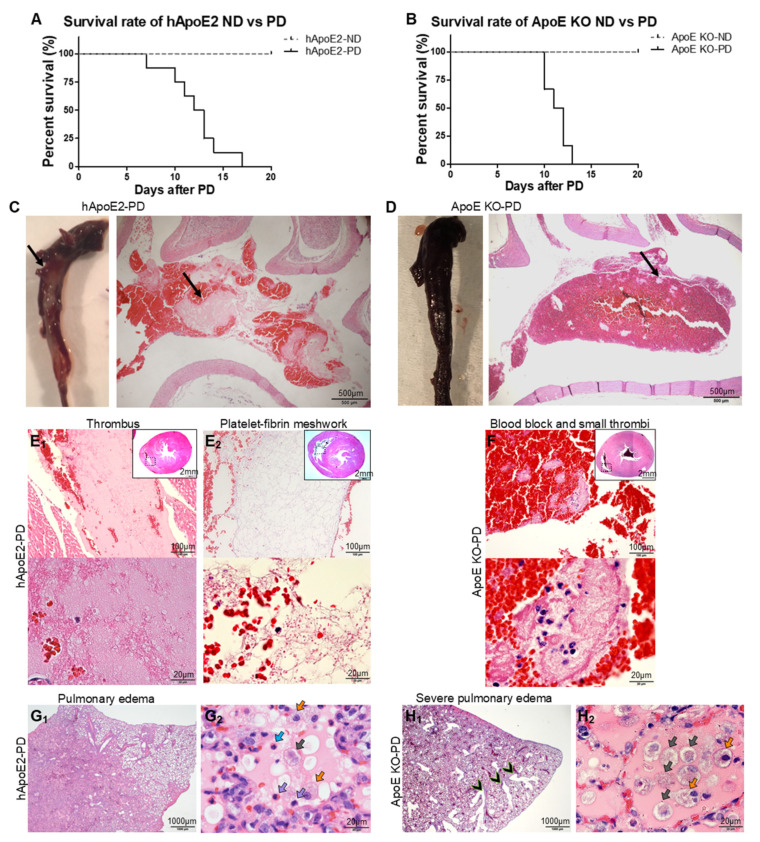
Survival rates and possible reasons for the death of hApoE2-PD and ApoE KO-PD rats. (**A**,**B**) The survival rates of hApoE2 and ApoE KO rats fed with the ND or PD are plotted and compared (hApoE, n = 8 each group; ApoE KO, n = 6 each group). (**C**) White color fatty embolus was detected in formalin-fixed hApoE2-PD aorta from autopsy (left gross image). Such fatty embolus was HE stained after paraffin preparation, shown in the right photomicrograph image. Black arrow indicates the fatty embolus in the aorta. (**D**) ApoE KO-PD aorta full of blood clot was HE stained. Blood cells were red stained, and small emboli were pink stained (black arrow). (**E_1_**,**E_2_**) Representative right ventricles of hApoE2-PD rats were HE stained. (**F**) Representative right ventricle of ApoE KO-PD rat was HE stained. Representative HE-stained slices of the hApoE2-PD (**G_1_**,**G_2_**) and ApoE KO-PD (**H_1_**,**H_2_**) lungs are shown. Neutrophils (blue), monocytes (purple), type II pneumocytes or alveolar macrophages (orange), and cell debris (gray).

As potential animal models for studies of atherosclerosis, the hApoE KI and ApoE KO rats were fed with an atherogenic PD [35]. PD feeding for a short period led to the premature death of all the hApoE2 and ApoE KO rats. The survival time is shown in Figure 6A,B. hApoE2 rats started to die from day 7, and all eight rats died after 17 days of PD treatment. All six ApoE KO rats died between 10 and 13 days of PD treatment. The death was determined mostly by in-cage death; only two deaths of hApoE2 rats were judged as reaching the humane endpoint by veterinarians. Due to the sudden death, the investigation on the metabolic profiles of PD-fed hApoE2 and PD-fed ApoE KO rats was incomplete.

We tested the lipid levels of two hApoE2 rats fed with the PD for two weeks and found that the hApoE2-PD rats had a 4 times higher TC level (~1700 mg/dL), 5 times higher TG (~2500 mg/dL), 22 times higher HDL-C (~230 mg/dL), and 9 times higher LDL-C&VLDL-C (~1000 mg/dL). Such surged blood lipid levels within a short period fostered the development of fat emboli in their vessels (Figure 6C aorta; Figure S2A small vessels in the lung, heart, liver, and kidney). Some fat emboli even completely occupied the vascular cavities (Figure S2A, hApoE2 lung, heart, and kidney). HE staining revealed an optical empty space which was caused by lipid dissolution during the staining process, dense fibrinoid material, and blood cells composed of the embolus of the hApoE2 aorta (Figure 6C). Autopsy of the PD-fed ApoE KO rats identified only blood block with sporadic small emboli in the aorta (Figure 6D), with increased lipid deposition in the small vessels of the hApoE2-PD lung, heart, liver, and kidney (Figure S2A). A dense fibrinoid thrombus (Figure 6E_1_) and loose platelet–fibrin meshwork (Figure 6E_2_) could be found in the right ventricles of hApoE2-PD rats. Meanwhile, autopsy of the PD-fed ApoE KO rats only demonstrated blood clots mixed with small thrombi in their right ventricles (Figure 6F).

Pulmonary edema was observed in both PD-fed hApoE2 (Figure 6G_1_) and ApoE KO rats (Figure 6H_1_). Edema fluid filled only partial lung lobes of PD-fed hApoE2 rats, while pulmonary edema spread to the entire lung of PD-fed ApoE KO rats. During this process, PD-fed ApoE KO rats developed severe bronchiectasis in a short period (Figure 6H_1_). The alveolar lumina of PD-fed hApoE2 and ApoE KO rats were filled with immune cells and mucus. Vacuole-like lipid droplets, pink-stained fluid, neutrophils, monocytes, type II pneumocytes or alveolar macrophages, and cell debris were detected in the alveolar lumen of hApoE2-PD rats (Figure 6G_2_). The alveolar lumen of ApoE KO-PD rats was full of pink-stained fluid, type II pneumocytes or alveolar macrophages, cell debris, and a few vacuole-like lipid droplets (Figure 6H_2_). Most of the immune cells were macrophages (Figure S2C,D). An empty space due to lipid dissolution after paraffin preparation in HE staining was observed in the alveolar lumen of PD-fed hApoE2 rats (Figure 6G_2_, Figure S2B).

PD-fed ApoE KO rats did not develop fat embolism blocking the aorta or vessels in their organs as PD-fed hApoE2 rats did. However, they developed much more severe pulmonary edema. Therefore, the cause of death of the PD-fed hApoE2 rats might be the formation of vascular fat embolism, while severe pulmonary edema could be the cause of the sudden death of the ApoE KO-PD rats.

### 2.7. Metabolic Profiles of WT, hApoE3, and E4 Rats after 4-Month PD

Four-month PD feeding significantly increased the bodyweight of WT, hApoE3, and hApoE4 rats (Figure S3A–C), with slightly decreased food consumption (Figure S3D–F). The fat mass of the WT, hApoE3, and hApoE4 rats was compared between rats fed with the ND or the PD for 16 to 18 weeks (Table 1). The mass of brown adipose tissue in WT rats was significantly higher after 4-month PD feeding. The mass of the inguinal subcutaneous and epididymal fats of the hApoE4 rats was significantly higher after PD feeding. The adipocyte size did not change after 4-month PD feeding (data not shown).

**Figure 7:**
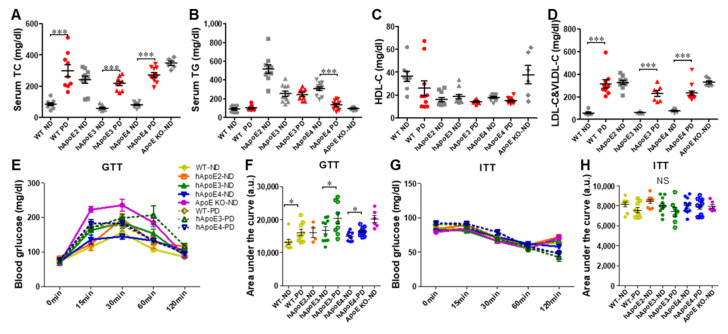
Metabolic changes in WT, hApoE3, and hApoE4 rats with 4 months of the PD. (**A**–**D**) Serum lipid levels were analyzed at age 6 months with or without PD feeding (TC and TG: WT n = 9 vs. 9, hApoE2 n = 10, hApoE3 n = 11 vs. 9, hApoE4 n = 11 vs. 11, and ApoE KO n = 6; WT n = 8 vs. 9, hApoE2 n = 10, hApoE3 n = 11 vs. 7, hApoE4 n = 11 vs. 12, and ApoE KO n = 6). (**E**,**F**) GTT was performed on rats at age 6 months (WT n = 8 vs. 9, hApoE2 n = 4, hApoE3 n = 10 vs. 9, hApoE4 n = 9 vs. 12, and ApoE KO n = 6). (**G**,**H**) ITT was performed on rats at the same age (WT n = 8 vs. 9, hApoE2 n = 9, hApoE3 n = 11 vs. 7, hApoE4 n = 11 vs. 11, and ApoE KO n = 6). (Two-way ANOVA followed by a Bonferroni post hoc test. *, *p* < 0.05, ***, *p* < 0.001; NS, no significant difference).

In addition, serum TC and LDL-C&VLDL-C were significantly increased after 4-month PD feeding, accompanied by slightly decreased HDL-C (Figure 7A–D). Only hApoE4-PD rats showed significantly decreased TG levels (Figure 7B). PD feeding impaired the glucose tolerance of WT, hApoE3, and hApoE4 rats in GTT (Figure 7E,F) but did not influence their insulin response in ITT (Figure 7G,H).

**Figure 8:**
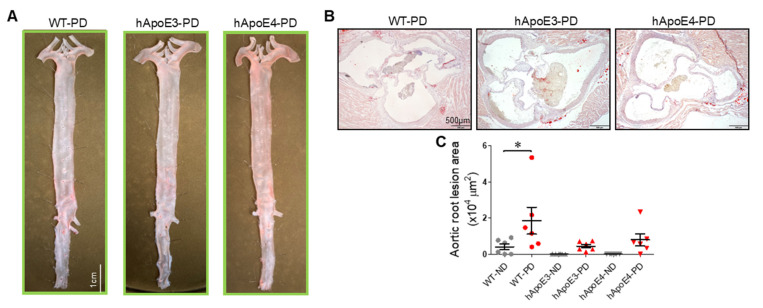
Atherosclerotic lesion after 4 months of the PD. (**A**) Representative photographs of Oil Red O-stained aortas from WT, hApoE3, and hApoE4 rats after 4-month PD feeding are shown. (**B**) Representative photomicrographs of the plaque lesion in cross-sectional aortic roots are shown. (**C**) Plaque lesion areas of cross-sectional aortic roots of these rats were quantified. (n = 6 per group, two-way ANOVA followed by a Bonferroni post hoc test. *, *p* < 0.05).

No obvious aortic plaques were detected in the en face aortas of the PD-fed WT, hApoE3, and hApoE4 rats (Figure 8A). Only mild atherosclerotic lesions were shown in their aortic roots (Figure 8B,C). Lesions in the hApoE3-PD and hApoE4-PD rats were even lighter than those in the WT-PD rats.

### 2.8. Thickening of Small Arteries in hApoE4 Rats Fed with 4-Month PD

**Figure 9:**
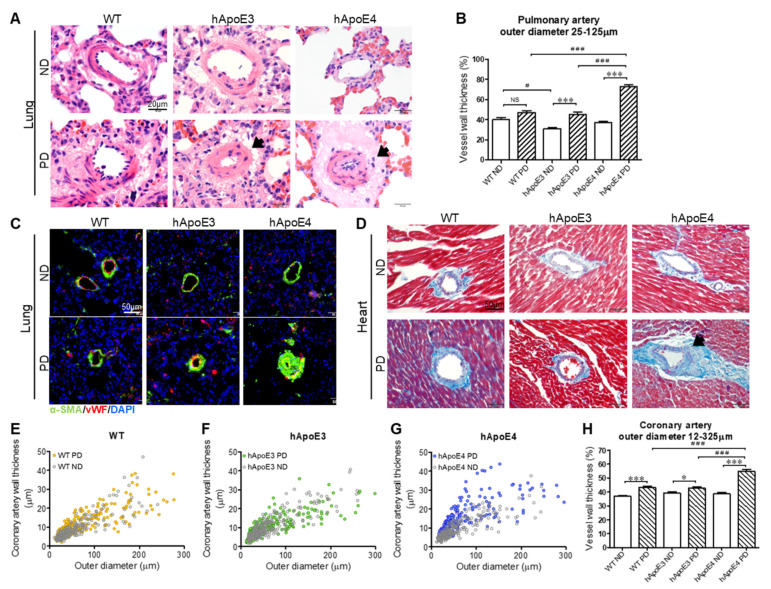
Vessel thickening of hApoE4 rats after PD. (**A**) HE-stained small arteries of rats fed with 4-month PD. The thickening of the small vessels is indicated with black arrows. (**B**) Statistical analysis of arterial wall thickness in lungs is shown. (n = 50–100 vessels from 9–11 rats for each group.) (**C**) The layers of vascular smooth muscle cells were stained by anti-α-SMA antibody. Endothelial cells were stained by anti-vWF antibody. Nuclei were stained by DAPI. (**D**) Masson-stained coronary arteries are shown. The thickening of coronary arteries is indicated by black arrow. Peri-coronary artery fibrosis was blue stained. (**E**–**G**) The thicknesses of coronary arteries with different outer diameters are compared between ND and PD groups. The arteries were imaged from the heart cross-sections at the papillary muscle level. (**H**) Wall thickness of coronary arteries is compared between WT, hApoE3, and hApoE4 rats fed with 4-month ND or PD. (n = 250–350 vessels from 9–11 rats for each group. Two-way ANOVA followed by a Bonferroni post hoc test. * and ^#^, *p* < 0.05; *** and ^###^, *p* < 0.001; NS, no significant difference).

The PD challenge caused vascular remodeling in hApoE4 rats. Obvious vascular remodeling of small arteries presented in the hApoE4-PD lung (Figure 9A), heart (Figure 9D), and liver (data not shown). In the lung and heart, 4-month PD feeding led to mild thickening of small arteries in the WT and hApoE3 rats, while the thickening of the vessel walls in the PD-fed hApoE4 rats was severer (Figure 9B,H). Occlusion of the vessel lumen in pulmonary arteries was found in the hApoE4-PD lungs (Figure 9A,C). PD-induced vascular remodeling was associated with the thickening of the vascular smooth muscle cell layers (Figure 9C).

There is a robust association of the ApoE4 isoform with an increased risk of CHDs [36,37]. As shown in Figure 9D–H, the coronary arteries of the PD-fed WT, hApoE3, and hApoE4 rats showed thickening of the vessel walls. The thickening of the coronary arteries in the PD-fed hApoE4 rats was much more severe than in the PD-fed WT and hApoE3 rats (Figure 9E–H). Additionally, peri-coronary artery fibrosis was observed in the Masson-stained cross-section of the hApoE4-PD hearts (Figure 9D, black arrow). Such peri-coronary artery fibrosis was not seen in the PD-fed WT and hApoE3 rats.

## 3. Discussion

In this study, hApoE2 rats developed spontaneous hyperlipidemia. The high TC and LDL-C&VLDL-C levels in the serum of hApoE2 rats, as well as the low HDL-C levels, predict their high atherosclerosis risk compared to WT rats, while the TC and LDL-C&VLDL-C levels of hApoE3 and hApoE4 are similar to those of the WT rats. The plaque formations in hApoE KI rats show the same trend as their blood atherogenic lipid levels. Sparse atherosclerotic lesions were found in the aortic roots of hApoE2 rats, but not in those of the hApoE3 or hApoE4 rats. Such plaque lesions in the hApoE2 rats were less severe than that in the ApoE KO rats with the regular diet. The short period of PD feeding killed the hApoE2 and ApoE KO rats, but not the hApoE3 or hApoE4 rats. Obvious vascular remodeling was found in hApoE4 rats fed with 4-month PD. Our results reveal that polymorphic human ApoE proteins have diverse influences on lipid profiles and plaque formation, and their impacts on vascular remodeling in response to the PD are also different in rat models.

The blood lipid changes in the hApE KI rats were quite similar to those of hApoE KI mice [18,19,20,38]: both hApoE2 rats and mice showed an atherosclerotic lipid profile with higher TC and TG, as well as lower HDL-C (at age 6 months); both rats and mice expressing hApoE3 or hApoE4 showed relatively normal lipid levels with no obvious plaques in vessels. The only difference regarding blood lipid levels is that the hApoE3 and hApoE4 rats showed a significantly lower HDL-C level in comparison to WT rats, while hApoE3 and hApoE4 mice had similar HDL-C levels to WT mice [18,20]. The hApoE2 rats developed more atherosclerosis in their aortic roots than the hApoE4 rats, which is different from the conditions in humans. Generally, the association with atherosclerosis and CVDs in humans is stronger in ApoE4 instead of ApoE2 carriers [13,14,15,16]. Recently, a meta-analysis revealed that ApoE4 increases the risk for heart disease [39], while ApoE2 increases the risks of peripheral artery disease, arterial aneurysm, and arterial thromboembolism [40,41]. The effects of ApoE2 on the onset of atherosclerosis remain uncertain because of the relative rarity of homozygotes in the population. Although a study reported that hApoE4 impaired macrophage efferocytosis in mice [42], the direct and precise impacts of ApoE2 and ApoE4 on atherosclerosis and the underlying mechanisms still need more investigation. Comparing with ApoE KO rats, hApoE2 rats developed milder plaque lesions in the aortic roots. Whether this is caused by the differences in their blood lipids needs further investigation. Overall, hApoE2 rats could be a good model for the investigation of special types of dyslipidemia, such as hypertriglyceridemia.

Different ApoE isoforms may have unique expression profiles in different tissues. The ApoE expression level in the plasma and livers of hApoE2 mice was higher [19,20,38,43], while we only observed a higher expression of ApoE in the livers of the hApoE2 rats. A different expression level of total ApoE has been reported in the plasma of individuals with different ApoE isoforms as well: plasma ApoE expression is slightly higher in ApoE2 carriers and lower in ApoE4 carriers [44,45]. The increased plasma ApoE in hApoE2 mice and ApoE2 carriers is possibly due to the higher synthesis rates of the proteins in the liver, as most of the blood ApoE proteins are secreted from the liver [46]. As ApoE2 shows an impaired binding affinity to receptors conducting the clearance of triglyceride-rich lipoproteins, the higher expression level of the ApoE protein in the hApoE2 rats may be due to a consequence of the feedback effect in which ApoE2 is over-produced by the liver of hApoE2 rats in order to compensate its impaired function. Additionally, we observed cirrhosis-like remodeling in the liver of the hApoE2 rats, which is independent of the diet type. The reason for the high ApoE level in the hApoE2 liver and its relationship with the hepatic nodular cirrhosis found in the hApoE2 rats is unknown and warrants further investigation. Another difference from the hApoE KI mice is in the bodyweight of the hApoE KI rats. We observed a significantly lower bodyweight in the hApoE2 rats with a lower white fat content and smaller adipocyte size than those of the hApoE3 and hApoE4 rats, while hApoE KI mice had a similar bodyweight with a slightly higher white fat content and larger adipocyte size in hApoE2 mice [38,47,48]. In a study of hApoE2 mice [38], ApoE2 proteins had a higher expression level in adipocytes, yet with a higher degradation rate. Lower triglyceride synthesis and elevated triglyceride hydrolysis were observed in adipocytes expressing ApoE2 in this study. The newly synthesized ApoE2 protein is unstable in adipocytes and may lead to defective lipogenesis and increased triglyceride hydrolysis. Interestingly, transplantation of adipose tissue from hApoE2 mice into hApoE3 mice led to a significantly decreased adipocyte size in the same study. Therefore, the smaller size of rat hApoE2 adipocytes could have several causes: (1) defective triglyceride internalization to cells due to the poor binding affinity of triglyceride-rich lipoproteins to the surface receptors conducting their clearance/internalization (accumulation of triglycerides in the serum reflects this); (2) defective fatty acid uptake through influx mechanisms of fatty acids in adipocytes; (3) decreased adipocyte triglyceride synthesis; and (4) increased triglyceride hydrolysis. The impaired lipid storage in the adipocytes might contribute to the lower fat mass of the hApoE2 rats. It was noticed that ApoE KO mice with defective lipid metabolism showed a preventive effect on obesity development [49]. Therefore, it is worth examining whether the ApoE2 isoform could prevent the development of obesity as well.

hApoE2 and ApoE KO rats developed only mild atherosclerosis. Therefore, we used the auxiliary PD and tried to generate atherosclerosis in these rats. The PD we used contains additional cholesterol (1.25%) and cholic acid (0.5%) in comparison to the Western diet, which is a promising tool to generate diet-induced atherosclerosis. Unfortunately, none of the hApoE2 and ApoE KO rats endured the challenge of the PD. The PD we used contained 21.2% of fat by weight (40 kcal% fat), the same as the Western diet. One study using a PD containing 15% of fat successfully induced coronary atherosclerosis in an ApoE KO rat model [50]. The high fat content in our PD might be the reason for the death of the hApoE2 and ApoE KO rats. However, their responses to the PD were different. The suspected cause of death of the hApoE2-PD rats is fat embolism found in the aorta and vessels in their organs, which could completely block the blood flow to vital organs, such as the brain, kidney, heart, liver, and lung. Meanwhile, the suspected cause of death of the ApoE KO-PD rats is acute pulmonary edema accompanied by bronchiectasis and considerable macrophage accumulation in the lung, which could lead to respiratory failure. A report of post-traumatic blood lipid changes stated that trauma patients, including patients with post-traumatic fat embolism, had a slow increase in the blood triglycerides level [51]. However, it is unknown whether the high serum triglyceride of the hApoE2 rats plays a role in the formation of fat emboli in their aortas and vessels in different organs after PD feeding. This is the first time that diet-induced fat embolism has been reported in an animal model. Therefore, the hApoE2-PD model can be used as an alternative tool for screening the therapeutic strategies of fat embolism.

In humans, there is a strong association of the ApoE4 isoform with an increased risk of CVDs, especially CHD and myocardial infarction [36,37,52]. CHD is the narrowing or blocking of the coronary arteries and is usually caused by atherosclerotic plaques. Most studies indicated that the increased risk of CHD is accompanied by increased levels of cholesterol, triglyceride, and LDL-C [53,54,55]. Little is known as to why the CHD incidence is higher in ApoE4 carriers. Additionally, no previous study had demonstrated the association of the ApoE4 isoform with CHD in animal models. We observed thickening of the coronary arteries and increased peri-coronary artery fibrosis in the hApoE4 rats after 4-month PD, without atherosclerotic lesions. The thickening of arteries other than the coronary arteries implies the onset of CHD [56,57,58]. In our study, thickening of the vascular walls in the lung and liver was observed in the hApoE4-PD rats as well (liver data not shown). In the pulmonary arteries, the PD-fed hApoE4 rats exhibited clear thickening of the vascular smooth muscle layer and occlusion of the vessel. Although both hApoE3 and hApoE4 rats developed modestly increased serum TC and LDL-C&VLDL-C after 4 months of the PD, the hApoE4-PD rats had much more severe wall thickening of the coronary arteries. The hApoE4 rats even developed a reduced TG level after PD feeding. Our data suggest that the ApoE4 isoform is a risk factor for CHD independent of high dyslipidemia, and that hApoE4-PD rats could be a potential model for vascular remodeling analysis.

## 4. Materials and Methods

### 4.1. Animals

All animal experimental procedures complied with the National Institutes of Health (NIH) Guide for the Care and Use of Laboratory Animals and were approved by the Institutional Animal Care and Use Committee (IACUC) at Georgia State University. Animals were kept on a 12:12 h light–dark cycle. The male Sprague Dawley WT rats (order code: 002), homozygous HsdSage:SD-*ApoE^em1Sage^* rats (ApoE KO, order code: 350), homozygous hApoE2 KI rats (hApoE2, order code: 394), homozygous hApoE3 KI rats (hApoE3, order code: 395), and homozygous hApoE4 KI rats (hApoE4, order code: 359) were from Envigo RMS (Saint Louis, MO, USA, previously named Horizon Discovery and SAGE Labs). All hApoE KI rats were generated by homology replacement of the endogenous rat ApoE gene with a different isoform of human ApoE [28].

### 4.2. Genotyping and Western Blotting

The genotyping protocol from Envigo RMS was used to identify WT, heterozygous, and homozygous animals. All the primers are listed in Table 2. Some of the hApoE KI rats were randomly selected and sequenced in the GSU core facility with 3730 Genetic Analyzers (Applied Biosystems, Foster City, CA) to confirm the ApoE isoforms. ApoE expressions in the liver, subcutaneous white fat, and serum were detected with Western blot using anti-human ApoE antibody (13366, Cell Signaling Technology, Danvers, MA, USA) and anti-rat ApoE antibody (ab183596, Abcam Cambridge, UK).

### 4.3. Diet, Bodyweight, and Food Consumption

Male rats, 8 weeks of age, were fed with normal diet (ND, 10 kcal% Fat, 0% Cholesterol, 0% Cholic Acid. D12109C, Research Diets Inc., New Brunswick, NJ, USA) or atherogenic PD (40 kcal% Fat, 1.25% Cholesterol, 0.5% Cholic Acid. D12109C) for 16 to 18 weeks (~4 months). WT, hApoE KI, and KO rats were randomly selected and grouped into ND and PD groups. A total of 10 groups were prepared, in which each group contained 9 to 11 rats, except the KO groups (6 rats). Bodyweight and food intake of rats were checked every week.

### 4.4. Lifespan

The lifespan of WT, hApoE KI, and KO rats fed with ND or PD was monitored. In-cage death of each animal was recorded, except two deaths of hApoE2 rats which were judged to reach the humane endpoint determined by a veterinarian in the animal facility.

### 4.5. Lipid Measurement

All lipid measurements were performed with rat serum collected at age 8 weeks and 24 weeks. Specifically, after overnight (~18 h) fasting, animals were restricted with a nose cone holder (Kent Scientific Corporation, Torrington, CT, USA), and blood samples were collected. After soaking the tail in warm water for 2 to 3 min, the lateral tail veins were well dilated and suitable for manipulation. About 0.2 mL of blood was obtained per animal with a 23 G needle. Serum was collected after 30-min coagulation at room temperature followed by a 10-min 3000× *g* centrifugation at 4 °C. Serum was stored at −80 °C until use. Reagents used to detect total cholesterol (TC) and total triglyceride (TG) were from Thermo Fisher Scientific (Waltham, MA, USA): Infinity™ Cholesterol Liquid Stable Reagent (catalog TR13421) and Infinity™ Triglycerides Liquid Stable Reagent (catalog TR22421). Cholesterol (catalog 23-666-198) and triglyceride (Catalog 23-666-422) standard solutions were from Thermo. TC and TG were detected with enzymatic colorimetric methods using ten times PBS-diluted serum. HDL-C and LDL-C&VLDL-C were measured using HDL and LDL/VLDL Quantitation Kit (catalog MAK045, MilliporeSigma, Burlington, MA, USA). Lipids levels were measured according to the manufacturer’s instructions. Briefly, serum was diluted four times with PBS first. HDL-C fraction and LDL-C&VLDL-C fraction were separated, following the detections of total cholesterol and free cholesterol in each fraction by using fluorometric assays. Concentration of cholesteryl esters in each lipoprotein was calculated by subtracting the free cholesterol value from the total cholesterol value. Serum samples to be compared were processed and detected at the same time to limit the operation/assay bias.

### 4.6. Blood Glucose Tests

Glucose level was measured by ReliOn glucose meter with a drop of fresh blood (~20 µL) from the tail vein. A 23 G needle was used to puncture the dilated lateral tail vein. After overnight (~18 h) food withholding, fasting glucose level was tested. Following overnight fasting, glucose tolerance test (GTT) was performed on rats administered with 2 g glucose per kg of bodyweight (2 g/kg BW) via i.p. injection. Blood glucose levels were detected at 0, 15, 30, 60, and 120 min relative to glucose administration. Insulin tolerance test (ITT) was carried out at least 48 h after the GTT, in which 0.75 U/kg BW insulin was used with i.p. administration after 6 h of fasting. Blood glucose levels in ITT were tested at the same time points as in GTT. Glucose or insulin was prepared in sterilized saline.

### 4.7. Histology

At age 6 months, animals were euthanatized with the carbon dioxide inhalation method followed by cervical dislocation according to the GSU IACUC Policy. The animal was transcardially perfused with 0.1 M phosphate-buffered saline (PBS, pH 7.4) for at least 5 min (~20 mL/min) or until the liver was cleared of blood. The organs and tissues were dissected and collected for the experiments. The weights of organs and tissues were recorded. Dissected tissues were fixed in 10% formalin solution for at least 3 days, following overnight preservation in 30% sucrose prepared with PBS at 4 °C. The tissues were frozen in OCT and cut into 8 µm sections with a cryostat. After air drying for 20 min, slices were washed with distilled water followed by 100% propylene glycol. Lipids on the slices could be stained by a filtered and pre-heated 0.5% Oil Red O solution prepared with propylene glycol at 60 °C for 30 min. Then, 85% propylene glycol was used to wash away the extra dye. Slices were counterstained with hematoxylin (H) solution and mounted in an aqueous mounting medium (H-5501, Vector Laboratories, Inc., Burlingame, CA, USA) and were imaged immediately with an Olympus BX53 microscope.

For Oil Red O staining of the aortic root, sequential cross-sections through the entire aortic valve were collected [59]. The 10 frozen sections with ~180 µm intervals were collected on each slide covering a distance of ~2 mm. Atherosclerotic lesion areas of aortic root were measured using Image J graphic Analysis System and were reported as the averaged percentage of lesion area (the Oil Red O-stained plaque area per aortic root area) from the serial 10 sections for each rat.

For Oil Red O staining of en face aortas, all adventitial fat was removed under a stereomicroscope. Each aorta was cut longitudinally and rinsed with distilled water followed by 60% isopropanol, then stained for 30 min in the freshly prepared Oil Red O working solution (0.3% Oil Red O prepared in 60% isopropanol, used within 24 h). After a quick wash with 60% isopropanol followed by distilled water, the aorta was pinned on the dissecting dish and imaged with a camera. Atherosclerotic lesion areas were measured.

Paraffin-embedded tissues were sectioned at a thickness of 5 µm and stained with the standard hematoxylin and eosin (HE) method or Masson’s trichrome stain kit (HT15-1KT, Sigma-Aldrich, St. Louis, MO, USA). After slices were mounted with Permount mounting medium (P15, Thermo Fisher Scientific, Waltham, MA, USA), images were captured. The size of the inguinal subcutaneous adipocytes of each rat was measured with Image J. At least 3 images were obtained from different adipose sections per animal.

For immunofluorescence of paraffin slices, an antigen retrieval step was performed before staining. Pulmonary macrophages were stained with anti-CD68 monoclonal antibody (ab31630, Abcam), followed by the corresponding secondary antibodies conjugated with Alexa Fluor 488. Macrophages were quantified as the percentage of total CD68+ cells. Anti-α-smooth muscle actin (α-SMA) (ab7817, Abcam) and anti-Von Willebrand factor (vWF) (ab11713, Abcam) antibodies were used to detect vascular smooth muscle cells and endothelial cells, respectively, followed by the corresponding secondary antibodies. Anti-GAPDH (sc 32233, Santa Cruz Biotechnology, Dallas, TX, USA) and anti-transferrin (17435-1-AP, Proteintech, Rosemont, IL, USA) antibodies were used as reference markers of tissues and serum, respectively. Slices were mounted with ProLong™ Gold Antifade Mountant with DAPI (P36935, Thermo Fisher Scientific). At least 3 images were obtained from different lung sections per animal.

### 4.8. Randomization/Double Blind

The animals assigned into ND or PD groups were randomly selected from the same species. The measurement of adipocyte size, measurement of plaque lesion areas, and cell counting of pulmonary macrophages were conducted double-blindly by two persons.

### 4.9. Data Analysis

All data were expressed as the mean and standard error of the mean (SEM). Statistical analyses were performed with Prism (GraphPad Software Inc., San Diego, CA, USA). For comparison between rat groups, a one-way ANOVA followed by a Tukey post hoc test was used. For analysis of the PD impacts, a two-way ANOVA followed by a Bonferroni post hoc test or Student’s t-test was used. Differences with *p* < 0.05 were considered significant.

## 5. Conclusions

This study reported the lipid metabolism and vascular histology features of hApoE KI rats. Although the phenotypes identified in the hApoE KI rats do not fully recapture the features of the carriers with polymorphic ApoEs, they are still valuable tools to study the functions of polymorphic ApoE proteins and their disease associations. In sum, hApoE2 rats could be a good model for studies of hypertriglyceridemia and the associated diseases, whereas hApoE4 rats could be a novel model for the research of vascular remodeling and CHDs in response to risk factors.

## Figures and Tables

**Table 1:** Weight of adipose tissues.

	Brown Fat (g)			Inguinal Subcutaneous Fat (g)			Epididymal Fat (g)		
	*Average*	*n*	*SEM*	*Average*	*n*	*SEM*	*Average*	*n*	*SEM*
WT-ND	0.59	9	0.05	9.53	9	0.62	7.96	9	0.53
WT-PD	0.77 *	9	0.05	9.86	9	0.6	10.51	9	1.07
hApoE2-ND	0.46	9	0.04	5.4	9	0.3	4.33	9	0.32
hApoE3-ND	0.56	11	0.04	10.17	11	0.83	7.43	11	0.56
hApoE3-PD	0.66	9	0.03	11.72	9	0.86	9.05	9	0.76
hApoE4-ND	0.44	10	0.03	8.93	10	0.81	8.13	10	0.61
hApoE4-PD	0.51	11	0.03	11.17 *	11	0.71	12.93 ***	11	0.82
ApoE KO-ND	0.59	6	0.05	10.02	6	0.84	8.19	6	1.22

*, *p* < 0.05; ***, *p* < 0.001. Student’s *t*-test, comparing to ND group with the same genotype.

**Table 2:** Primers used for genotyping.

Primers	Sequence (5′-3′)	WT Band	E2/3/4 Band	KO Band
Rat ApoE F	CGAGGGAGAGCTGGAGGT	150 bp		134 bp
Rat ApoE R	TGTGTGACTTGGGAGCTCTG			
Human ApoE F	CTGGAGGAACAACTGACCCC		437 bp	
Human ApoE R	CTGCCCATCTCCTCCATC			
5’ Cel-1 F	CACCCCGGGGTGCTGAGATAGAGAT	455 bp		455 bp
5’ Cel-1 R	TTCCACCATGTTGGGCTCCG			
3’ Cel-1 F	GTGCGCTCCAAGATGGAGGA	388 bp		388 bp
3’ Cel-1 R	GGCCATGGAATGTGTGCTATGTC			

## Data Availability

Data are available from the corresponding author on reasonable request.

## Supplementary Materials

The following are available online at https://www.mdpi.com/article/10.3390/ijms22158262/s1.

## Abbreviations

CVDs	cardiovascular diseases
CHD	coronary heart disease
AD	Alzheimer’s disease
WT	wild-type
ApoE	apolipoprotein E
ApoE KO	ApoE knockout
hApoE KI	humanized ApoE knockin
PD	Paigen diet
ND	normal diet
TC	total cholesterol
TG	total triglyceride
GTT	glucose tolerance test
ITT	insulin tolerance test
