# Supplementary material for: Features of Lipid Metabolism in Humanized ApoE Knockin Rat Models
Source: Int J Mol Sci. 2021 Jul 31;22(15):8262. doi: 10.3390/ijms22158262 (PMC8347964; doi:10.3390/ijms22158262)

Supplementary figures

Characterization of Lipid Metabolism in Humanized ApoE Knockin Rats

Yang Wu[1], Gem Johnson[1], Fujie Zhao[1], Yin Wu[1], Guojun Zhao[2], Andrew Brown[2], Shaojin You[1], Ming-Hui Zou[1] and Ping Song[1],*

[1] Center for Molecular and Translational Medicine, Georgia State University, Atlanta, GA, United States
[2] Envigo RMS, Inc., St. Louis, MO, United States
* Correspondence: psong@gsu.edu; Tel.: 001-404-413-6636; Address: Center for Molecular and Translational Medicine, Georgia State University, 157 Decatur St SE, Atlanta, GA 30303

Supplementary figure S1. No obvious ectopic lipid deposit was found in heat, liver, and glomeruli of hApoE2 rats fed with ND. A-C. Oil red O stain was used to assess the histological visualization of neutral fat deposits in different tissues with 8 μm frozen sectioning. No obvious abnormal lipid deposit was detected in the heart, liver, and glomeruli in all rats. **D.** Gross liver images of WT, hApoE KI, and ApoE KO rats were shown. The white arrow indicates the hepatic nodular tissue of hApoE2 rats. White dashed box indicated the normal tissue. **E.** Representative HE stained photomicrographs of liver sectioning were from the areas of the livers (white dashed boxes in **D**). The hepatocytes were arranged into strips and radiated outwards from a central vein. In the area (white dashed box) other than nodular area (white arrow) of hApoE2 liver, hepatic structure was normal.

Supplementary figure S2. Increased lipid deposits and tissue damages were found in organs of hApoE2 and ApoE KO rats fed with PD. A. Increased lipid deposit was detected in organs of hApoE2-PD and ApoE KO-PD rats with Oil red O stain (8 μm frozen sectioning). In the lung of the hApoE2-PD rat, macrophages and small vessels showed increased lipid deposits. In the lung of the ApoE KO-PD rat, alveolar fluid was stained by Oil red O dye. Both hApoE2-PD and ApoE KO-PD rats had increased fat deposits in cardiomyocytes, branches of coronary arteries, hepatic cells, hepatic small vessels, glomeruli, and small vessels of the renal medulla. **B.** Fatty emboli (white arrow indicated fat vacuoles after paraffin preparation) were detected in hApoE2-PD rats neither ApoE KO-PD rats. **C.** Massive CD68-positive macrophages were detected in lungs of hApoE2-PD and ApoE KO-PD rats. **D.** Quantification of CD68-positive macrophages in the lung is shown. (Data was analyzed with 3-4 slices per rat and totally from 3-5 rats for each group. Two-way ANOVA followed by a Bonferroni post hoc test. $^{\#}$, $P<0.05$; ***, $P<0.001$.)

Supplementary figure S3. Four-months PD feeding increased the bodyweight of the rats, but the food consumptions were less. A-C. Bodyweights of WT, hApoE3, and hApoE4 rats were significantly increased with PD in comparison to those with ND. (WT: ND n=9, PD n=9; hApoE3: ND n=11, PD n=9; hApoE4: ND n=11, PD n=11. Two-way ANOVA. *, $P<0.05$; ***, $P<0.001$.). **D-F.** However, the food intakes per day of these rats were less with PD feeding.

Supplementary figure S1
Wu et al.

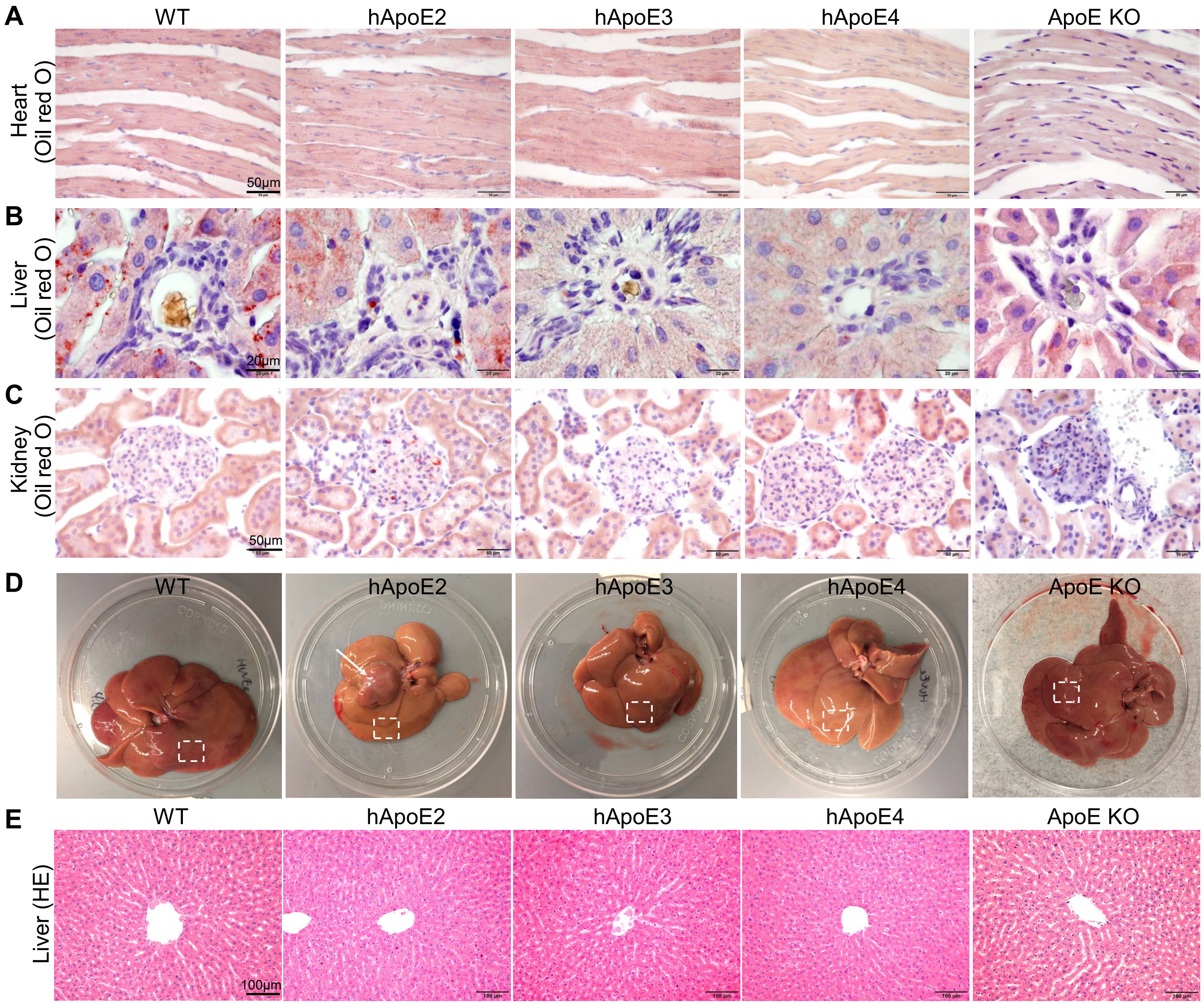

Supplementary figure S2
Wu et al.

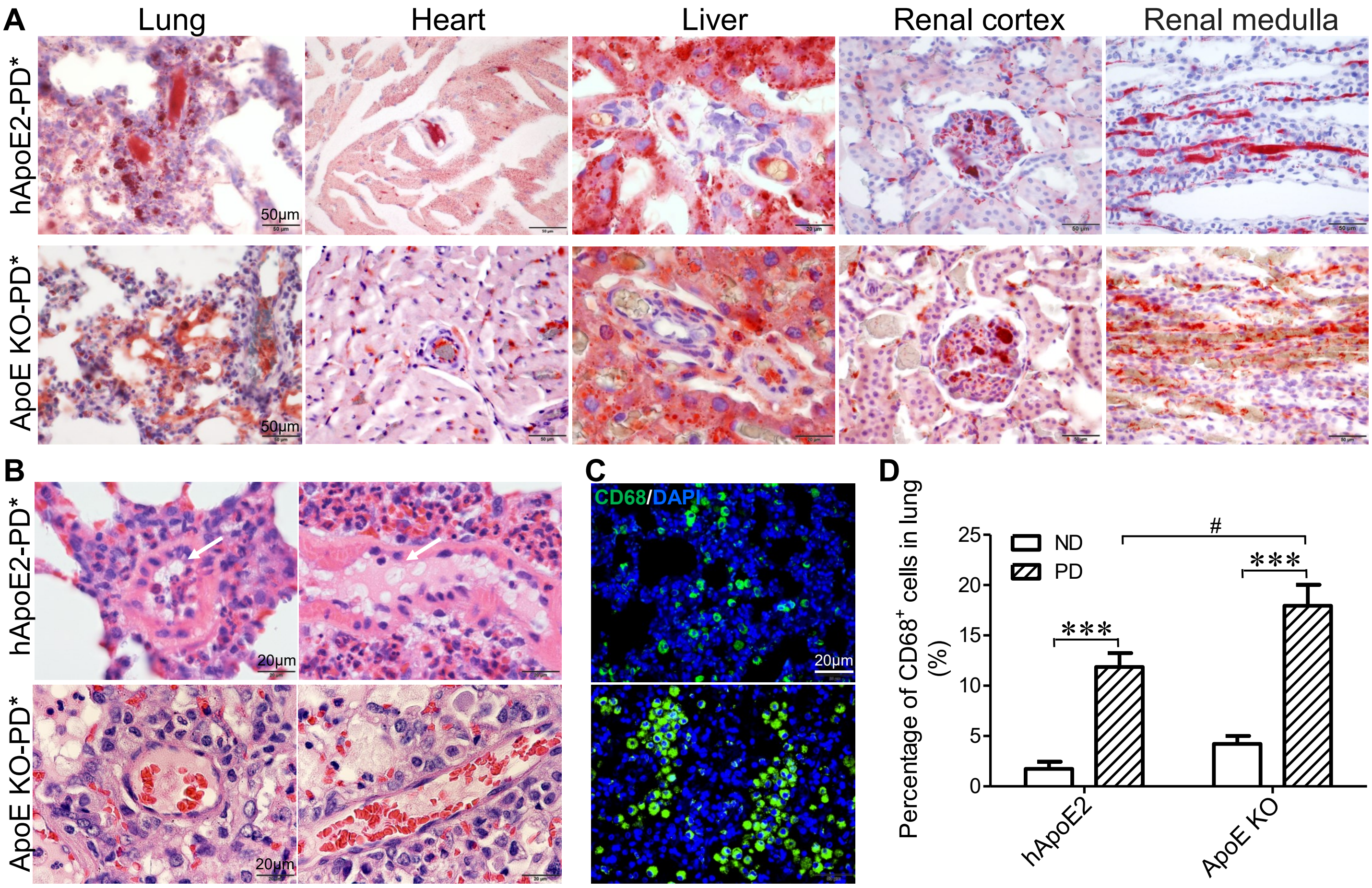

Supplementary figure S3
Wu et al.

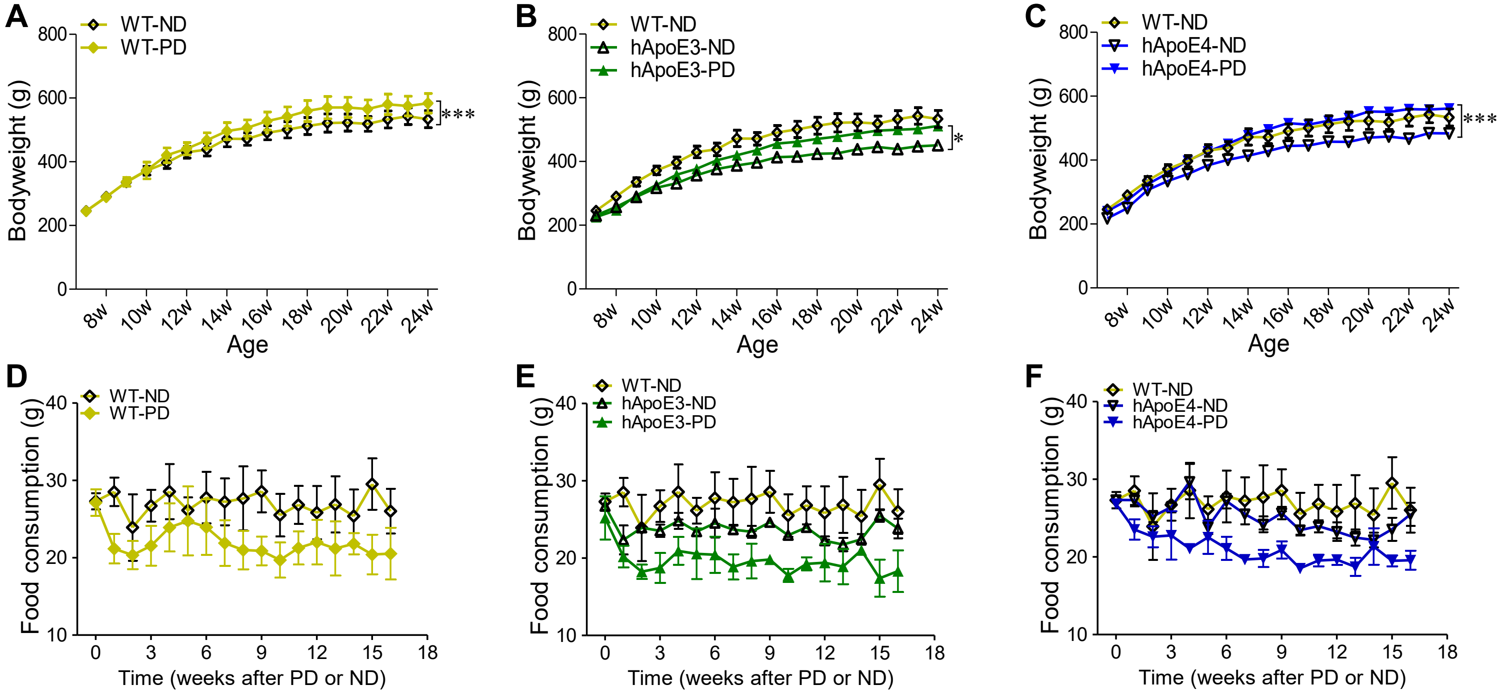

Supplement: Supplementary file 1 [file ijms-22-08262-s001.zip › ijms-1284271-supplementary.pdf]
